# Iliocaval Venous Obstruction, Cardiac Preload Reserve and Exercise Limitation

**DOI:** 10.1007/s12265-020-09963-w

**Published:** 2020-02-10

**Authors:** Rachael I. Morris, Paul A. Sobotka, Peter K. Balmforth, Eric J. Stöhr, Barry J. McDonnell, Darren Spencer, Gerard J. O’Sullivan, Stephen A. Black

**Affiliations:** 1 School of Cardiovascular Medicine and Sciences, King’s College London, London, UK; 2 The Ohio State University, Columbus, OH USA; 3 V-Flow Medical Inc., Saint Paul, CA USA; 4 Department of Medicine, Division of Cardiology, Columbia University Irving Medical Centre, New York City, USA; 5 Cardiff School of Sport and Health Sciences, Cardiff Metropolitan University, Cardiff, UK; 6 Galway University Hospitals, Galway, Ireland

**Keywords:** Venous stenosis, Venous obstruction, Iliac venous disease, Preload, Exercise capacity

## Abstract

Cardiac output during exercise increases by as much as fivefold in the untrained man, and by as much as eightfold in the elite athlete. Increasing venous return is a critical but much overlooked component of the physiological response to exercise. Cardiac disorders such as constrictive pericarditis, restrictive cardiomyopathy and pulmonary hypertension are recognised to impair preload and cause exercise limitation; however, the effects of peripheral venous obstruction on cardiac function have not been well described. This manuscript will discuss how obstruction of the iliocaval venous outflow can lead to impairment in exercise tolerance, how such obstructions may be diagnosed, the potential implications of chronic obstructions on sympathetic nervous system activation, and relevance of venous compression syndromes in heart failure with preserved ejection fraction.

## Introduction

Three main pathways of venous drainage exist to return deoxygenated blood to the heart: the superior vena cava, draining the upper limb and head and neck; the inferior vena cava (IVC), which receives drainage from the lower limb and pelvis via the iliac veins; and the portal vein, which receives blood from the gastrointestinal tract via the hepatic sinusoids and hepatic vein before draining to the IVC. Superior vena cava obstruction is well known to cause oedema of the upper extremities and dyspnoea due to decreased venous return. Obstruction of the portal vein is also known to have profound consequences including portal hypertension, ascites, and formation of varices. Chronic obstruction of the IVC, however, has traditionally been seen as a relatively benign condition, with ligation of the infra-renal IVC being the preferred treatment for recurrent pulmonary embolism until the introduction of IVC filters in the late 1960s. In this manuscript, we will discuss the important cardiovascular consequences of obstruction of the iliac veins and IVC (iliocaval veins) on preload reserve and exercise tolerance, as well as potential implications of venous compression in other cardiac disorders.

## Preload Reserve and Venous Return

**Fig. 1:**
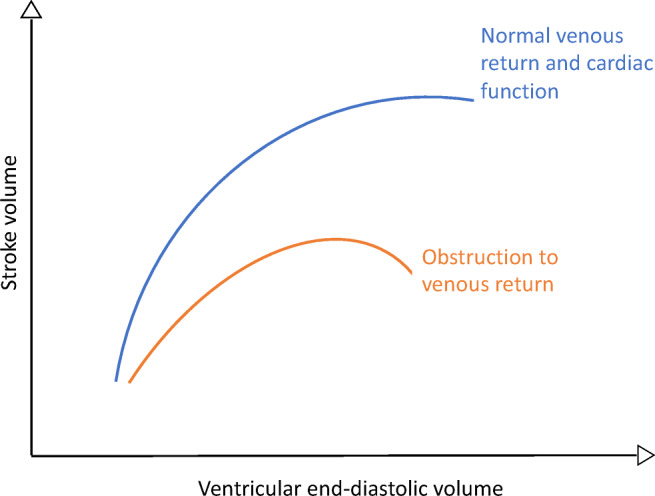
A graph depicting Frank-Starling relationship between ventricular end-diastolic volume and stroke volume in the normal heart, and in preload impairment secondary to venous obstruction

Preload reserve describes the dependence of cardiac output on venous return to the heart, independent of heart rate and contractility, and is a critical feature of how the body responds to changes in posture and exercise. Increased heart rate, vasoconstriction, and cardiac inotropy are well-described features of the baroreceptor-mediated responses to standing and exercise; however, the principal mechanism for increasing stroke volume is best explained by the ventricular function curves initially described by Frank and Starling. At each level of ventricular function, an acute incremental increase of myocyte stretch, due to returning venous blood volume, results in increased stroke volume (Fig. 1). A notable exception is when the ventricle is on horizontal portion of the function curve, when incremental filling fails to increase stroke volume, presumably because of optimal myocyte overlap and stretch-related calcium metabolism. Starling’s work also highlighted the critical relationship between afterload and stroke volume. These relationships underscore the importance of venous return to increasing output by optimizing cardiac stretch. Whilst it is axiomatic that increasing preload drives the increases in stroke volume related to standing and exercise, less often recognized is that limitations on venous return may significantly limit the cardiovascular response to exercise too.

At rest, the venous system contains approximately 70% of the total blood volume [1], the majority of which does not generate a measurable pressure (the ‘unstressed blood volume’). The ‘stressed blood volume’ generates pressure and is the major contributor to venous return. Sympathetic activation causes venoconstriction, which can significantly increase the stressed blood volume. Animal studies have demonstrated that extreme sympathetic activation can result in as much as 18 ml/kg increase in stressed volume, an increase of more than 100% [2–6]. Equally, decrease in sympathetic activity leads to an increase in venous capacitance and unstressed volume [7–10].

It is known that at rest, 25% of the total cardiac output is distributed to the splanchnic bed [11], one of the largest reservoirs of unstressed blood volume. The splanchnic bed is richly innervated with adrenergic receptors which can respond rapidly to sympathetic stimulation [12]. Splanchnic venoconstriction is recognised as one of the most important reflex responses to increase central blood volume, preload and cardiac output upon standing; however, during sustained exercise, the majority of blood flow is directed to the lower limbs, and splanchnic blood flow is reduced to 5–10% of total cardiac output [11]. The increase in venous return during upright exercise must therefore come predominantly from the lower limb, which is dependent upon the functioning of several key components: hydrostatic height, cardiac ‘vis-a-tergo’ and ‘vis-a-fronte’ (forward pressure pushing blood from the veins towards the right atrium, and suction force from the right atrium pulling blood from the veins), peripheral muscle pumping action, venoconstriction and competency of venous valves [13]. Obstruction to the iliocaval veins, including compression and intraluminal pathologies (Table 1), can restrict outflow from the lower limb and impair venous capacitance, limiting the rate of flow back to the heart, in addition to causing venous hypertension and damage to valves.

**Table 1:** Causes iliocaval venous outflow obstruction

Venous compression	Intraluminal pathology
Pregnancy	Acute deep vein thrombosis
Obesity	Post-thrombotic syndrome
Pelvic or abdominal malignancies	IVC atresia/agenesis
Benign pelvic mass (e.g. fibroids)	IVC filter thrombosis
Enlarged lymph nodes	
Overlying iliac arteries—typically the right common iliac artery but may occur at any vessel crossing point	

## Physiological Changes During Exercise

Cardiac output increases during exercise by as much as fivefold in the healthy untrained man, and as much as eightfold in endurance athletes [14]. Whilst the cardiac output rises with exercise, brain blood flow remains constant and blood flow to the heart rises in response to cardiac metabolic demands. Exercising lower extremity muscle increases oxygen extraction as well as regional blood flow [15]. During maximal exercise in an untrained individual, mixed venous oxygen saturation falls from ~ 75% at rest to ~ 25–30% [15]. Unlike cardiac oxygen extraction, which is near maximal at rest, striated muscle increases its extraction during exercise [16, 17].

The lower extremity veins perform a dual role during exercise: to increase venous return to the heart enabling preload cardiac reserve, and to reduce the venous pressure in the lower limbs in the presence of significantly increased arterial blood flow. Thus, limitations of venous capacity may only be realized during exercise when the regional blood flow is increased. In this instance, exertional induced venous hypertension and oedema is a logical consequence. The venous hypertension and congestion may result in swelling of the extremity and exercise-induced pain (venous claudication) [18] not associated with arterial obstruction and ischemia. Limiting venous return during exercise can impede the capacity of the heart to increase perfusion to exercising striated muscle, causing exercise intolerance, associated with inadequate selective arterial perfusion during exercise, increased oxygen extraction and ultimately regional lactic acid production despite normal arterial anatomy and underlying ventricular function.

## Diseases Which Can Impair Venous Return to the Heart

A number of disorders can impair the preload reserve mechanisms, resulting in reduced exercise tolerance. For example, both constrictive pericardial diseases and restrictive myocardial disorders limit incremental preload as a mechanism of increasing stroke volume by shifting the pressure volume relationship of heart filling beyond compensatory capacity [19–21]. Similarly, pulmonary hypertension can prevent left ventricular filling despite adequate right heart volumes, and exercise tolerance is significantly impaired [22, 23]. In a different situation, namely the syndrome of pure autonomic failure (previously Bradbury Eggleston syndrome or idiopathic orthostatic hypotension), many of the autonomic nervous system mechanisms increasing venous return are lost. The presenting symptoms are most often orthostatic hypotension, with associated dizziness and light-headedness, on occasion precipitating orthostatic loss of consciousness. These autonomic syndromes differ from the mechanical venous obstruction by nature of associated symptoms of dry mouth, diarrhoea or constipation, changes in urinary habits, alterations of smell and erectile dysfunction. Patients with impaired venous return secondary to pulmonary hypertension, constrictive pericardial disease, restrictive myocardial disease or iliocaval venous outflow obstruction may all present with exertional dyspnoea; however, resting cardiac imaging would be expected to be normal if there is a peripheral rather than a cardiac cause of impaired preload. All patients may present with peripheral oedema; however, additional signs such as venous claudication, varicosities or skin changes may indicate some degree of venous outflow obstruction.

## Causes of Iliocaval Venous Obstruction

### Venous Compressions

Compression of the iliac veins can be caused by inherent anatomic features, such as overriding artery, ligament or bones, or acquired complications such as uterine fibroids [24]. Even fat in the confined space within the pelvis can compress the veins [25]. In a like manner, pregnancy may be associated with the transient compression of the iliocaval venous segment [26]. The anecdotal instructions of lying on the left side to avoid hypotension during pregnancy well demonstrate this potential. Other conditions such as post pelvic surgery and/or bowel surgery, whereby the iliocaval veins are displaced causing compression may interfere with venous capacitance [27].

The point at which the right common iliac artery crosses the left common iliac vein, usually the level of the L5 vertebrae, is a frequent location of anatomical venous compression. Virchow initially described this phenomenon in 1851 [28] as an explanation for the increased incidence of deep vein thrombosis in the left lower limb. May and Thurner later found a 22% prevalence of ‘venous spurs’ in the lumen of the left iliac vein in their study of 430 cadavers [29], and hypothesised that these were a result of the pulsations of the overlying artery causing compression against the lumbar vertebrae and chronic irritation of the vascular endothelium. More recent studies have suggested that a quarter of individuals have up to 50% compression of the left common iliac vein observed on cross-sectional imaging [30]; however, the true prevalence is not known as the majority are asymptomatic, and there is a lack of population level data on the condition. Less commonly, compressions may also occur on the right-hand side and at the inferior vena cava.

**Fig. 2:**
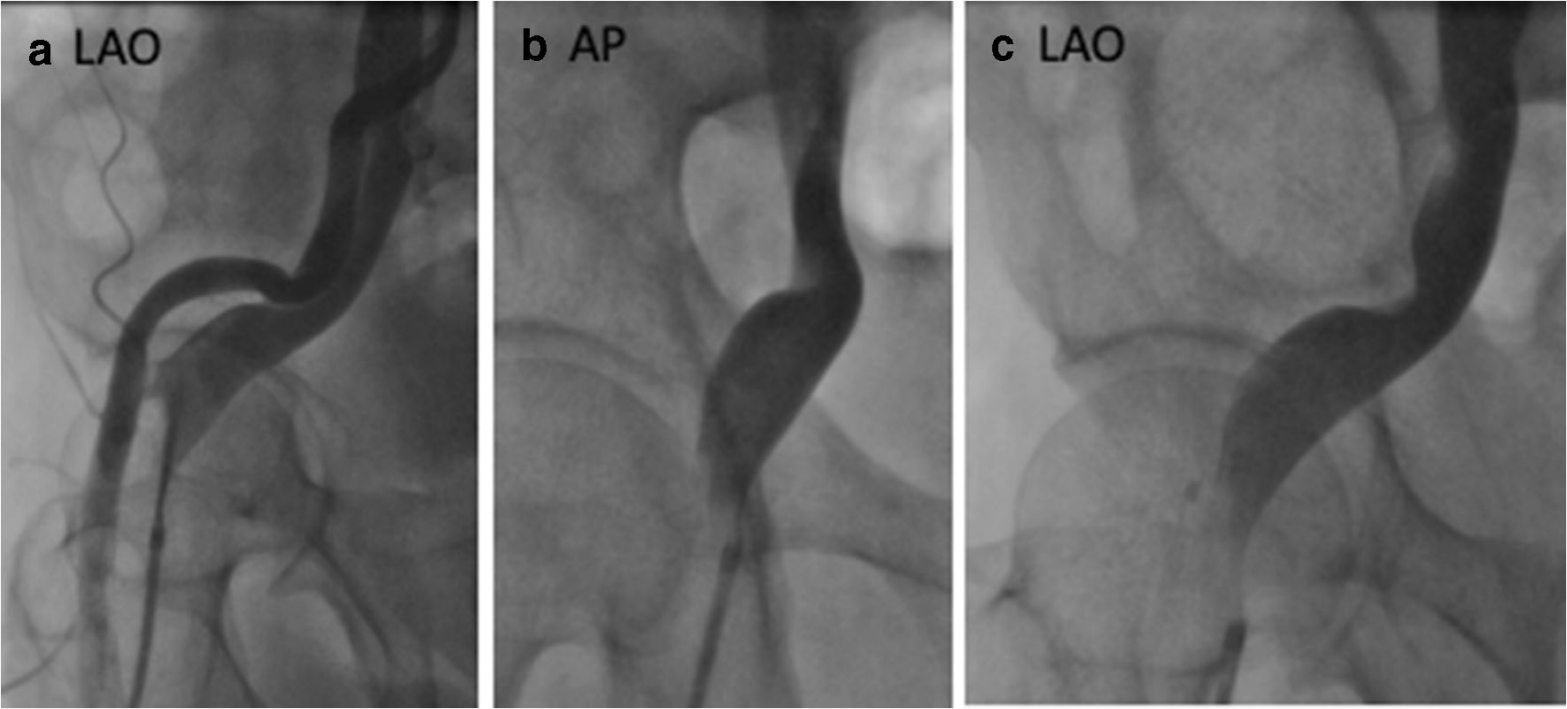
**a** Simultaneous arterial and venous contrast injection in a therapy resistant hypertensive patient, with no signs or symptoms of leg swelling (LAO). **b**, **c** Demonstrate impeded contrast flow in the vein via direct overriding arterial compression from both AP and LAO angles respectively

Arterial calcifications of overriding artery may convert benign intersections into pathological. Thus, arterial ageing itself may be a risk factor to acquiring venous disease. Potential mechanisms linking reduced venous return to overt arterial disease potentially relates to lower cardiac output and resultant lower baroreceptor activity. The reduced baroreceptor activity creates an exaggerated increase in sympathetic activity in order to increase arterial tone and maintain appropriate blood pressure needed to facilitate perfusion. However, this increased sympathetic activity has been shown to increase arterial remodelling and stiffening. Our group has already illustrated that increased aortic stiffness correlates with increased aortic calcification [31]. Furthermore, iliac arteries are associated with calcification deposition due to their proximity to the aortic bifurcation and resultant flow and pressure turbulence in this region. Therefore, lowering venous return through iliocaval outflow obstruction may potentially create a more rigid arterial segment close to the presenting venous region and turn those benign intersecting regions into pathological areas of overt disease progression (Fig. 2).

### Intraluminal Obstructions

Congenital variants of the IVC affect an estimated 4% of the population, and include interruption, hypoplasia, duplication or agenesis [32]. The most common presentation is of incidental finding on cross-sectional imaging; however, agenesis of the IVC is found in 5% of patients under 30 with deep vein thrombosis [33]. Obstruction of the iliofemoral veins and inferior vena cava can also be acquired as a consequence of deep vein thrombosis, in the acute phase with thrombus material occluding the lumen of the vessel, and in the chronic phase where there may be incomplete thrombus resolution leading to scar tissue and collagen formation. Deep vein thrombosis has a prevalence of 1 in 1000 [34], with a third of these involving the iliocaval veins. Despite adequate anticoagulation, up to 50% of patients will go on to develop post-thrombotic syndrome [35] as a result of chronic venous hypertension, and this proportion increases further in those patients with iliofemoral rather than femoro-popliteal venous thrombosis [36].

**Fig. 3:**
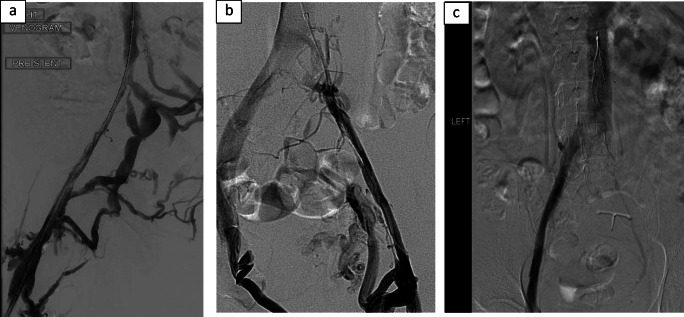
Contrast venography demonstrating venous collaterals in post-thrombotic syndrome (**a**, **b**). **c** May-Thurner syndrome in a patient with indwelling IVC filter

**Fig. 4:**
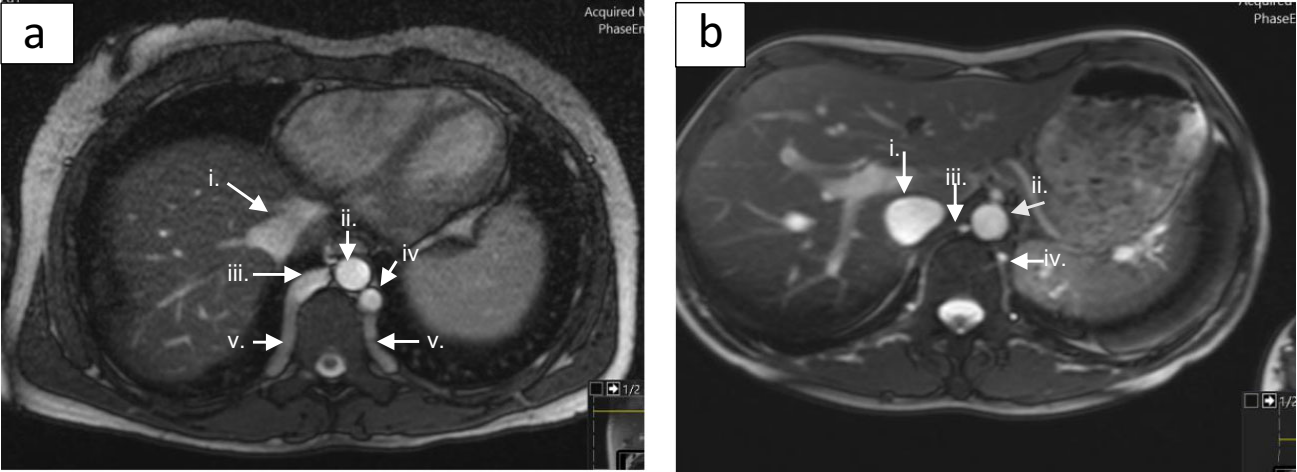
MRV demonstrating hypertrophied azygos, hemiazygos and spinal veins in a patient with previous DVT affecting IVC and iliac veins (**a**) compared with normal venous anatomy (**b**). **(**i) IVC, (ii) aorta, (iii) azygos vein, iv. hemiazygos vein v. spinal collaterals

When there is significant venous outflow obstruction, flow may be diverted through collateral pathways. These collaterals may be evident in the thigh, groin or abdominal wall, and can cross to the contralateral side (Fig. 3). In IVC outflow obstruction, hypertrophy of the azygous, hemiazygos, lumbar and spinal veins is commonly seen (Fig. 4). The ability of these collateral networks to accommodate increased venous return may be sufficient at rest, but their fixed diameter may limit venous return during exercise.

With the main return pathway of the venous circulation occurring via the iliofemoral veins and the IVC, it would be illogical to assume that a fixed obstruction of these vessels is without haemodynamic consequence in any individual. The reduction of total venous return has pathological consequences which may immediately not be known due to the absence of symptoms or may be attributed to pre-existing conditions such as shortness of breath, decreased exercise tolerance and leg pain during exertion.

### Impaired Exercise Tolerance in Patients with Iliocaval Venous Obstruction

Exercise intolerance attributed to reduction in venous return from the lower limbs was first described by Varat et al. in 1970 [37], who reported results from exercise right heart catheterisation in 5 patients who had previously undergone ligation of the inferior vena cava, and developed exertional dyspnoea post-operatively. None of the patients had any evidence of pulmonary hypertension. Increase in cardiac index at increasing levels of exercise was significantly less than that in controls, reflected in the exercise factor (increase in cardiac output per minute per 100-ml increase in oxygen consumption). Patients with IVC ligation had a mean exercise factor of 406-ml increase in cardiac output per 100-ml increase in oxygen consumption (280–493 ml) vs mean 726-ml increase (569–822 ml) in controls. At rest, there was no significant difference in cardiac output between the groups.

Reduced venous return to the heart as a cause of exercise intolerance in patients with IVC ligation was suggested again in study by Miller and Staats [38], who reported cardiopulmonary exercise testing with gas exchange analysis in four patients. All four achieved < 64% of expected oxygen consumption, with appropriate increases in heart rate and relatively normal gas exchange.

Ben Dov et al. [39] reported a case of a 63-year-old male with bilateral iliofemoral and inferior vena cava thrombosis who experienced symptoms of dyspnoea, dizziness and leg pain upon walking a few steps on ground level. Extensive investigation for cardiac and respiratory causes of exercise intolerance revealed normal cardiac function with no evidence of ischaemia, and a mild restrictive pattern on pulmonary function tests which could not account for the disability. Imaging revealed complete occlusion of the inferior vena cava and left iliac vein, with severe stenosis of the right iliac vein. Cardiopulmonary exercise testing was carried out for upper extremity and lower extremity cycle ergonometry and found normal peak VO2 for upper extremity exercise (1.8 L/min) but severely reduced peak VO2 during lower extremity exercise (1.25 L/min). The authors concluded that the collateral venous drainage through moderately enlarged paravertebral veins to the azygous system was not adequate under the condition of high leg flow demand, thereby limiting the rise in cardiac output.

This group later conducted cardiopulmonary exercise testing in 9 patients with chronic iliofemoral vein outflow obstruction (IFVO) as a result of previous DVT, comparing results between upper and lower limb cycle ergonometry, to 11 healthy controls, and a separate experiment comparing lower limb CPET results in controls with and without application of thigh tourniquets [40]. All patients had previous investigations to rule out chronic thromboembolic pulmonary hypertension and had normal lung function tests. Patients with IVFO achieved a significantly lower percentage of predicted peak VO2 (median 50% predicted, range 36–83%) compared to controls (median 88% predicted, range 67–129%), and had a higher ratio of median arm: leg peak VO2 (0.95, range 0.77–1.43 vs 0.73, range 0.6–1.0). Application of one thigh tourniquet inflated to 30–40 mmHg in controls reduced median VO2 max to 81% predicted (63–110%), and with two tourniquets this reduced further to 76% predicted (55–108%). We contacted the authors of the study, but they were unable to provide us with data on the individual VO2 max values achieved by patients. Data on cardiac output, arterial saturations, lactate, ventilatory slopes and skeletal muscle oxygenation would be of value to determine the physiology behind reduced exercise capacity in the patient group to strengthen the authors’ conclusion that peripheral limitation to exercise can result from venous occlusion.

## Diagnosis of Iliocaval Venous Obstruction

Iliocaval venous obstruction should be considered a differential diagnosis in patients presenting with reduced exercise tolerance or exertional dyspnoea. A thorough history is essential and must include details of any previous thrombotic events, family history of thrombosis and previous treatment for chronic venous insufficiency such as ulceration or varicose veins. Physical examination should include inspection of the lower limbs for peripheral oedema, skin changes such as lipodermatosclerosis, hemosiderin deposition, venous eczema, ulceration (active or healed) and varicosities, which may also be visible in the pelvis and abdominal wall.

Imaging of the venous system with duplex ultrasound and cross-sectional abdominal and pelvic imaging (MRI or CT) in the first instance should be undertaken for patients with suspected iliocaval venous pathology. Duplex ultrasound should be a first-line investigation and can identify reflux, flow abnormalities and presence of collaterals in the lower limb. Positional changes in flow can also be identified, and dynamic assessments can be performed with the patient standing, and during Valsalva, which may reveal abnormalities that were not apparent at rest. Due to their deep location, identification of compression or intraluminal obstruction of the iliocaval veins is less reliable with ultrasound, particularly in patients with larger body habitus. MRI or CT venography can be used for more detailed visualisation of the abdominal and pelvic veins; our group prefers MRI as it reduces radiation exposure avoids the need for intravenous contrast agents. Invasive assessment with contrast venography has long been considered gold standard for identification of venous outflow obstruction and visualisation of collateral pathways; however, intravascular ultrasound has more recently been demonstrated to have a superior sensitivity and specificity for identification of iliac vein lesions [41].

These modalities can be used to identify the presence of an obstructive lesion with a reasonable degree of accuracy; however, the presence of obstruction alone does not necessarily indicate significance. When making decisions about treating patients with venous outflow obstruction, clinicians have tended to use a value of 50% stenosis or more coupled with clinical symptoms as indication for treatment [42]. However, with the exception of duplex ultrasound, all of the current techniques visualize the venous system whilst the patient is resting and in the supine position. Resting venous capacitance may not reveal the demands of increased venous volume during exercise. The increases of lower extremity arterial blood flow by up to three–four times the resting consumption [43] require an identical venous capacitance; thus, resting venous anatomy may conceal limited venous capacitance during exercise. Increases of venous pressure or limitations of preload reserve during exercise may be critical in identifying underlying venous disease. Dynamic assessment of venous flow during exercise is therefore necessary to fully determine the haemodynamic significance of an obstructive lesion [44], in addition to measures of right atrial filling and cardiac output.

## Potential Relevance in Heart Failure with Preserved Ejection Fraction

Patients with heart failure with preserved ejection fraction (HFpEF) may have normal stroke volume at rest, but they are unable to sufficiently increase cardiac output to sustain exercise. The underlying pathophysiology of exercise intolerance in HFpEF is poorly understood, and likely multifactorial [45]. Several mechanisms have been proposed, including diastolic dysfunction, abnormal ventricular-arterial coupling, inflammation and endothelial dysfunction and depressed heart rate response [46].

Activation of the sympathetic nervous system is known to occur as a compensatory mechanism to overcome low stroke volume in patients with heart failure [47–49], including increased activation of the renin-angiotensin-aldosterone pathway, increased plasma epinephrine and norepinephrine and stimulation of adrenergic receptors. Dysregulation eventually leads to sympathetic overdrive and disease acceleration. Sympathetic activity in patients with iliocaval outflow obstruction has not yet been investigated; however, it is entirely plausible that compensation for low stroke volume would occur through a similar mechanism in this patient group. It is also conceivable therefore that a venous compression causing impaired preload could be a contributing factor in ventricular remodelling and HFpEF pathophysiology. Interestingly, both HFpEF and left iliac vein compression syndrome (May-Thurner syndrome) are more common in females, with 30–40% higher prevalence of HFpEF [50–52], and May-Thurner syndrome being twice as common in females [53]).

Peripheral oedema and venous congestion are typically considered consequences of advanced cardiac failure, but there is evidence that endothelial damage and inflammation are in themselves associated with increased activity of the sympathetic nervous system [54]. Venous congestion and oedema are well-described symptoms of venous hypertension [55]; therefore, patients with obstruction to venous return from the lower limb may develop increased activation of the sympathetic nervous system as a result of endothelial damage, in addition to compensatory activation to overcome reduction in preload (Fig. 2). Columbo and co-workers [56] created a model of venous hypertension using unilateral tourniquet inflation and measured the resulting release of inflammatory mediators, neurohormones and activation of endothelial factors. Plasma interleukin-6, endothelin 1, angiotensin II, vascular cell adhesion molecule 1 and chemokine ligand d2 were all significantly increased in the congested arm compared to the contralateral arm. These findings suggest venous obstruction and resulting interstitial oedema may participate in development of both regional and systemic inflammation and its consequences.

## Conclusions

Obstruction of the iliocaval venous outflow may lead to reduction in venous return from the lower limb during exercise, reduced stroke volume and subsequent reduction in cardiac output. Iliocaval outflow obstruction may be diagnosed as an incidental finding on cross-sectional imaging, or in patients with overt lower limb symptoms; however, resting studies may not fully reveal the haemodynamic significance of an obstructive lesion in relation to venous return to the heart. Given the prevalence of venous obstruction in the population, further research studies are needed to quantify the impact of such pathology on cardiac function during exercise, and to explore the potential for venous hypertension and lower extremity oedema to cause systemic sympathetic activation and chronic inflammation.
